# Expanding clones, expanding aneurysms through macrophage-to-osteoclast differentiation

**DOI:** 10.1172/JCI206748

**Published:** 2026-04-15

**Authors:** Jessica A. Regan, Svati H. Shah

**Affiliations:** 1 Division of Cardiology,; 2 Duke Molecular Physiology Institute, and; 3 Duke Center for Precision Health, Duke University School of Medicine, Durham, North Carolina, USA.

## Abstract

Abdominal aortic aneurysms (AAAs) are an age-related cause of sudden cardiac death and cardiovascular disease (CVD) morbidity with limited nonsurgical treatment options. In this issue of the *JCI*, Yonekawa et al. addressed the pathobiologic mechanisms of clonal hematopoiesis (CH), the age-related acquisition of expanded somatic clones in blood cells, as a potential driver of AAA. CH prevalence was high in patients being treated for AAA, and faster AAA expansion occurred over a period of one year in CH carriers. In an angiotensin II–induced model of AAA, mice carrying ten-eleven translocation 2 (*Tet2*) mutations (*Tet2*-CH) displayed accelerated AAA development and macrophage reprograming to an osteoclast-like state. Inhibition of this differentiation, targeting RANK/RANKL with FDA-approved therapies like alendronate and denosumab, suppressed aneurysmal growth. These findings suggest that macrophage-to-osteoclast differentiation may underlie the risk and progression of AAA associated with age-related CH, a mechanism that is modifiable through existing therapeutics.

## Expanding from germline to somatic drivers of cardiovascular disease

With advances in next-generation sequencing technology, clonal hematopoiesis (CH) has been identified as an age-related cardiovascular disease (CVD) risk factor (1). CH is the age-related accumulation of driver mutations in myeloid cells, most commonly in the epigenetic modulators *DNMT3A* and ten-eleven translocation 2 (*TET2*), which occur in the absence of hematologic abnormalities and can be detected by genetic sequencing of PBMCs (2). CH is uncommon in young patients, but the prevalence rises to greater than 10% in individuals aged 65 years or older, making this a relatively common disease marker of aging. Since seminal work in 2017 that used both human and murine data to establish the relationship between CH, heightened levels of systemic inflammation, and atherosclerotic CVD (1, 3), rapid investigation in this space has extended the associations of CH to include heart failure, atrial fibrillation, as well as noncardiac disease states (4), although the full mechanistic architecture remains to be articulated.

## Evidence for CH as an AAA risk marker

**Figure 1:**
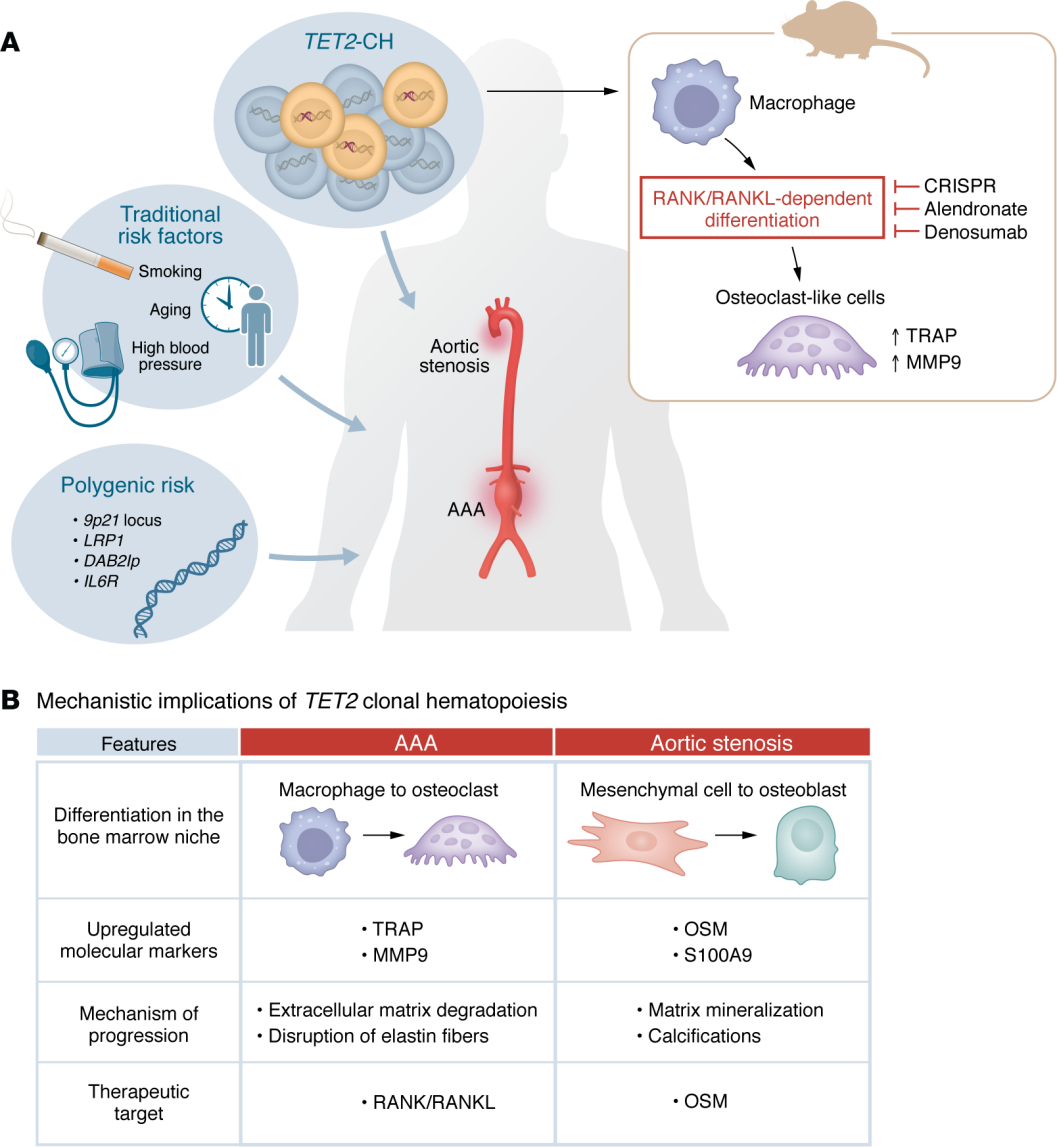
Expanding CH clones promote aneurysm growth via macrophage-to-osteoclast–like differentiation. (**A**) Yonekawa et al. (7) identified a mechanism of *TET2*-CH–driven AAA via RANK/RANKL-dependent macrophage-to-osteoclast–like immune cell differentiation. Aortic macrophages isolated from *Tet2*-CH mice were characterized by upregulation of TRAP and MMP9 expression, and both the TRAP^+^ differentiation and the aneurysm phenotype in *Tet2*-CH mice could be inhibited by genetic or pharmacologic targeting of RANK/RANKL. (**B**) Distinct mechanisms of *TET2*-CH–driven immune cell differentiation have been described for AAA by Yonekawa et al. (7) versus aortic valve stenosis by Abplanalp et al. (12). While Yonekawa et al. (7) found osteoclast-like differentiation to cause extracellular matrix degradation in AAA, Abplanalp et al. (12) described paracrine effects of OSM and S100A9 to stimulate mesenchymal cell–to-osteoblast transitions contributing to matrix mineralization and calcifications.

Importantly, abdominal aortic aneurysms (AAAs) remain a major, silently progressive, age-related CVD with high rates of mortality and limited nonsurgical treatment options. Prior human studies established an association between CH and ICD code-defined thoracic aortic aneurysm or AAA (5). These findings extended the known nongenetic and polygenic germline drivers of AAA to now include somatic mutations as potential contributors to aneurysm risk (Figure 1A).

Known pathologic features of traditional aneurysm risk include loss of smooth muscle cells, extracellular matrix degradation, and infiltration of immune cells (6). However, despite epidemiologic evidence linking CH with aneurysm risk, there remains a gap in the mechanistic understanding of this risk. In this issue of the *JCI*, Yonekawa et al. addressed this gap by pairing longitudinal, ultradeep sequencing and aneurysm imaging in humans with murine models of *Tet2*-driven CH (hereafter referred to as *Tet2*-CH) to identify dysregulated immune cell differentiation (7).

First, bone marrow with 20% *Tet2*-mutant cells was transplanted into apolipoprotein E–deficient (*Apoe*-deficient) mice. *Tet2*-CH was selected as the model of choice, given the predominant inflammatory immune cell phenotype that has been described for *TET2*-CH in humans (1, 3). Flow cytometry displayed expansion of *Tet2*-mutant cells in peripheral blood, mimicking the phenomena of CH observed in humans. There were no overt aortic abnormalities at baseline, however, after 4 weeks of angiotensin II infusion to induce AAA, *Tet2*-mutant mice had larger aneurysms with disrupted elastic fibers on histology, despite increases in blood pressure that were comparable to that seen in wild-type controls.

To further support the pathogenic role of CH in AAA expansion, the authors performed deep sequencing of 17 genes (including *TET2*) in peripheral blood from 44 patients with known AAA undergoing endovascular aneurysm repair at a single center in Japan. This deep, targeted sequencing technology identified a much higher (60%) prevalence of CH than previously reported in epidemiologic cohorts using whole-exome or whole-genome sequencing (10%–20%) for patients approximately 70 years of age (8). Here, Yonekawa et al. considered very small clones, with a variant allele fraction (VAF) under 2%. Individuals harboring these small clones comprised the majority of individuals with CH identified in the study. Importantly, there was no statistical difference in age in the CH group compared with the group without CH, in contrast to studies using the definition of CH of indeterminant potential (CHIP) requiring a VAF of 2% or higher (1). The authors next looked retrospectively at imaging obtained one year prior to AAA stent graft placement in 31 patients and found that patients with CH had greater aneurysm growth than did those without CH (4.3 ± 2.8 vs. 2.8 ± 1.3 mm/year, *P* = 0.04). There was no association between the rate of aneurysm growth and VAF, a finding discordant with other CHIP studies in which large clones (VAF ≥10%) have been described to confer greater CVD risk (9, 10).

## Clasts versus blasts as mechanistic links

Having established a pathologic link between CH and AAA progression in both mice and humans, the authors then examined immune cell changes in mice following angiotensin II infusion and found increases in bone marrow–derived CCR2^+^ macrophages as mediators of accelerated aneurysm development. These findings suggest that *Tet2*-mediated alterations in myeloid cell fate may contribute to AAA pathogenesis. RNA-seq of macrophages isolated from aortic tissue of *Tet2*-mutant mice identified upregulation of tartrate-resistant acid phosphatase type 5 (*Trap*) and elevated *Mmp9* mRNA expression as markers of bone resorption and extracellular matrix remodeling. TRAP, a marker of activated osteoclasts, has been previously identified in aneurysmal tissue, suggesting that osteoclast-like cells may be a source of proteases that contribute to aortic tissue instability (11). In the present work by Yonekawa et al., stimulation with osteoclastogenic receptor activator of NF-κB ligand (RANKL) drove greater TRAP and MMP9 expression in *Tet2*-mutant mice. This mechanistic link adds substantiating evidence to the proposed mechanism of how expanding clones skew immune cell differentiation, triggering greater proteolysis-induced extracellular matrix and elastic fiber degradation, thereby contributing to expanding aneurysm growth.

Early atherosclerotic models of murine *Tet2*-CH identified upregulation of bone marrow–derived cytokines and chemokines, with heightened levels of IL-18 in humans harboring *TET2* mutations (1). Similar to aortic aneurysm management, aortic stenosis lacks biomarkers and noninvasive therapies for disease progression. In recent work, also published in the *JCI*, Abplanalp et al. found a greater prevalence of aortic stenosis in All of Us, BioVU, and UK Biobank participants with CH (12). To understand the mechanisms of this association, single-cell RNA-seq of PBMCs from patients with *TET2*-CH and aortic stenosis identified proinflammatory monocytes with expression of procalcific signaling factors, including oncostatin M (OSM) and osteoblastic differentiation. In vitro macrophage models of *TET2*-CH showed heightened expression of procalcific secreted inflammatory factors (OSM, IL-23, and S100A9) with paracrine effects triggering mesenchymal cell–to–osteoblast–like differentiation and greater calcific, mineralized deposits. *Tet2*-mutant mice were found to have greater aortic valve calcium deposition, and culture of their aortic valve tissue showed greater secretion of OSM and S100A9. Downregulation of OSM in culture ameliorated calcific cell phenotypes, suggesting a potential therapeutic target for calcific aortic valve disease. Taken together with the present work, distinct but related osteoclastic and osteoblastic mechanisms appear to underpin *TET2*-CH–induced aortic aneurysm growth via extracellular matrix degradation and calcific aortic valve stenosis via matrix mineralization (Figure 1B).

## Macrophage-to-osteoclast differentiation — a therapeutic target?

Finally, to explore the therapeutic potential of targeting macrophage-to-osteoclast differentiation, Yonekawa et al. leveraged a genetic knockout of RANK, as well as pharmacologic inhibition of osteoclast precursor differentiation with bisphosphonates or anti-RANKL monoclonal antibody (similar to the clinically used osteoporosis medication denosumab). *Tet2*-mutant/*Rank*–knockout mice displayed reductions in aneurysm size and elastic fiber histology as compared with *Tet2*-mutant/*Rank* wild-type controls. In vitro and in vivo treatment with the bisphosphonate alendronate inhibited TRAP^+^ differentiation and ameliorated the aneurysm phenotype in *Tet2*-mutant macrophages and mice, respectively. Administration of anti-RANKL antibody also reduced aortic diameters in *Tet2*-mutant mice compared with control mice. These findings highlight the pathogenicity of *Tet2*-CH–induced osteoclast-like differentiation in aneurysm growth and the therapeutic prospect of modulating this pathway to prevent aneurysm progression.

## Limitations, clinical implications, and future directions

The findings by Yonekawa et al. bridge clinical observations and translational science to identify a mechanism of CH-induced vascular disease while highlighting the potential of repurposing existing pharmacotherapies for CH-associated CVD. While the study advances our mechanistic understanding of CH in AAA, we must acknowledge that these findings may not be generalizable to thoracic or other aneurysmal beds. How much macrophage-to-osteoclast differentiation specifically contributes to AAA risk compared with other nonmacrophage mechanisms remains unclear. Furthermore, it is not clear that the mechanisms relevant in mice translate to the same pathobiologic mechanisms in humans, particularly given the need for angiotensin II exposure to induce AAA and the relatively higher percentage of *Tet2*-mutant cells present in the mouse model compared with what was observed in the human study. Finally, the retrospective nature of the human cohort introduces the possibility of reverse causation in an elderly, comorbid population with examination of very small CH clones.

Although AAAs are more prevalent in men, and women tend to present with AAAs 5–10 years later, women may present with more advanced disease and have a higher risk of rupture at smaller aortic diameters and higher perioperative mortality (13, 14). Therefore, as in other disease states and CVD broadly, it is critical that we study mechanisms of disease risk in both sexes. In the present work, the cohort was approximately 90% male, and only male mice were studied to establish the *Tet2*-CH driven macrophage-to-osteoclast differentiation. Therefore, future work should both strive for greater enrollment of female participants and verify these findings in female mice to ascertain common or distinct mechanisms across biological sex.

To date, inflammation has emerged as the key mechanistic link between CH and CVD across preclinical and clinical research. As proof of principle, secondary analyses from trials of the antiinflammatory therapeutic agents canakinumab (15) and colchicine (16–18) have shown potential benefit in participants with CH. As we await prospective CH clinical trials of antiinflammatory therapies, we must now in parallel consider CH as a biomarker for AAA progression in larger human studies. Incorporation of flow cytometric profiling in prospective human studies of patients with and without CH to identify TRAP^+^ osteoclast-like cells would confirm the translational mechanisms identified here, informing precision therapies targeting RANK/RANKL/TRAP-mediated macrophage differentiation.

Future work will need to examine if these mechanisms are *TET2*-CH specific. Interestingly, prior data support the role of *DNMT3A*-driven CH in osteoporosis with enhanced osteoclastogenesis that could also be ameliorated by alendronate or cytokine targeting, extending the generalizability of similar mechanisms to at least the two most frequently mutated CH genes (19). It remains unclear whether CH-driven macrophage-to-osteoclast differentiation is invoked in other CVD pathogenesis, including coronary atherosclerosis and heart failure, and this question deserves special attention in future work. Reassuringly, bisphosphonates and denosumab are already clinically approved therapies and could therefore be more easily repurposed to study their role in targeting osteoclast differentiation to potentially slow aneurysm progression.

## Conclusion

In summary, this *JCI* study adds a disease-specific mechanism to our growing understanding of the adverse effects of CH as an age-related biomarker. The study offers a conceptual model for how expanding somatic clones trigger immune cell differentiation from macrophages to TRAP^+^, tissue-destructive, osteoclast-like cells, disrupting elastin fibers and the extracellular matrix, thereby contributing to aortic aneurysm expansion. This creates a strong rationale for prospective studies to better understand and therapeutically target RANK/RANKL/TRAP-mediated macrophages in CH and perhaps in aortic aneurysms and CVD more broadly.

## Conflict of interest

SHS reports research funding through sponsored research agreements with Duke University from Astra Zeneca, Lilly Inc., Verily Inc., and nference and is a co-inventor on unlicensed patents held by Duke University.

## Funding support

This work is the result of NIH funding, in whole or in part, and is subject to the NIH Public Access Policy. Through acceptance of this federal funding, the NIH has been given a right to make the work publicly available in PubMed Central.

- NIH grant 1K38HL175026 (to JAR).

## Version 1 04/15/2026

Electronic publication
